# Engineered *E. coli* Nissle 1917 for delivery of bioactive IL-2 for cancer immunotherapy

**DOI:** 10.1038/s41598-023-39365-2

**Published:** 2023-08-02

**Authors:** Sarunas Tumas, Trine Sundebo Meldgaard, Troels Holger Vaaben, Sara Suarez Hernandez, Annemette Tengstedt Rasmussen, Ruben Vazquez-Uribe, Sine Reker Hadrup, Morten O. A. Sommer

**Affiliations:** 1 Novo Nordisk Foundation Center for Biosustainability, Technical University of Denmark, Lyngby, Denmark; 2 Department of Health Technology, Technical University of Denmark, Lyngby, Denmark

**Keywords:** Cancer, Protein delivery, Interleukins

## Abstract

In this study we performed a step-wise optimization of biologically active IL-2 for delivery using *E. coli* Nissle 1917. Engineering of the strain was coupled with an in vitro cell assay to measure the biological activity of microbially produced IL-2 (mi-IL2). Next, we assessed the immune modulatory potential of mi-IL2 using a 3D tumor spheroid model demonstrating a strong effect on immune cell activation. Finally, we evaluated the anticancer properties of the engineered strain in a murine CT26 tumor model. The engineered strain was injected intravenously and selectively colonized tumors. The treatment was well-tolerated, and tumors of treated mice showed a modest reduction in tumor growth rate, as well as significantly elevated levels of IL-2 in the tumor. This work demonstrates a workflow for researchers interested in engineering *E. coli* Nissle for a new class of microbial therapy against cancer.

## Introduction

In the last decades significant advances have been made with therapies that harness the immune system to fight cancer. Contrary to chemotherapy or radiotherapy, immunotherapy drugs are not directly toxic to cancer cells. Instead, these drugs activate the immune cells or direct them to recognize and kill cancerous cells. Because of their effectiveness, immunotherapy is rapidly becoming a powerful first-line treatment against cancer^1^. Interestingly, the origins of this treatment modality go back as far as the eighteenth century. The first reported observations were made after cancer patients with bacterial infections showed significant tumor regression^2^. These observations were followed by attempts to create bacterial-based therapies to treat cancer, but the results of these studies were inconclusive^3^. Although the concept of using bacteria to modulate the immune response was abandoned, these experiments provided the first crude demonstrations of an attempt to treat cancer by harnessing the immune system using bacterial-based therapies.

A recent resurgence in bacterial-based immunotherapy strategies has taken place after the observation that specific bacteria can selectively colonize tumors when injected intravenously^4,5^. Despite extensive preclinical evidence^6–8^, translation of bacterial-based immunotherapies into humans has not yet materialized. However, advances in synthetic biology provide the means of modifying these tumor-homing bacteria to enhance their capabilities for therapeutic applications.

The use of genetically modified microbes for therapeutic application, also called Advanced Microbial Therapeutics (AMTs), is a promising strategy for local delivery of bioactive molecules and proteins in the human body. Bacteria can colonize and live in different sites of the human body, such as the skin, gut and even tumors^9^. In that manner, *Salmonella typhimurium* has been extensively studied for production of bioactive compounds in the tumor microenvironment^10–13^. This is especially useful for local delivery of proteins with a short half-life or proteins that are too toxic to be used systemically. One such class of proteins are cytokines^14^.

Cytokines are small signaling proteins that help coordinate the immune response. They comprise a large family of proteins that are secreted by various types of cells and exert signaling effects on both immune cells and other cell types^14^. The cytokine IL-2 is a powerful growth factor of T cells and natural killer (NK) cells^15,16^. IL-2 stimulation is crucial for the activation and differentiation of T cells and NK cells towards a cytotoxic and effector phenotype. CD8^+^ T cells and NK cells are key effector cells that participate in direct killing of cancer cells. However, there is increasing evidence that CD4^+^ T cells also play a key role in supporting the antitumor activity of CD8^+^ T cells by producing cytokines required for their activation and differentiation, as well as directly killing cancer cells upon recognition of tumor antigens in the context of major histocompatibility II complex^17,18^. Therefore, IL-2 is a key stimulation factor to drive immune cell cytotoxicity towards cancer cells. However, clinical use of IL-2 is limited due to its high systemic toxicity, short half-life in plasma and poor penetration into the target area^19,20^.

Intratumoral IL-2 delivery using engineered microbes has been studied previously using *Salmonella typhimurium*^21^ *and Clostridium species*^22,23^*.* However, recent studies have shown that probiotic *Escherichia coli* Nissle 1917 (EcN) bacteria can also colonize tumors and may exhibit a better safety profile than *S. typhimurium*^24^. Furthermore, *E. coli* strains have been previously used to deliver therapeutic peptides in vivo in the tumor microenvironment^25–27^.

In this study we present an AMT designed for local delivery of IL-2 directly in solid tumors using the probiotic EcN. We performed strain optimization to increase bioactive IL-2 protein production outside of the cell. Several rounds of optimization were performed and the ability of the most promising candidate to stimulate cytotoxicity of immune cells was determined with a 3D tumor spheroid assay. Finally, the anticancer properties of the most promising strain were validated in vivo using a syngeneic CT26 colon carcinoma animal model. The results demonstrated the ability of the optimized strain to modulate the tumor-microenvironment by doubling the intratumoral levels of IL-2 and moderately slowed down the growth rate of tumors.

## Results

### Optimization of microbially produced IL-2 in EcN

**Figure 1:**
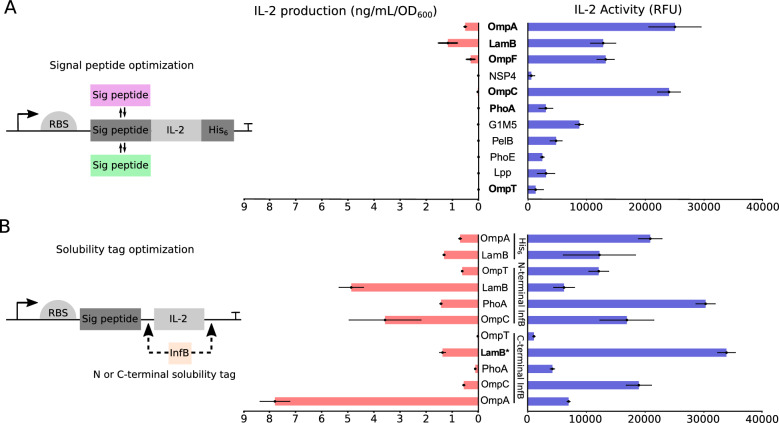
Optimization of signal peptide and solubility tag leads to higher cytokine production and activity in the supernatant. (**A**) Different signal peptides were cloned upstream of the IL-2-His_6_ gene. Bacterial supernatants were collected and IL-2 levels in the supernatant were measured using an IL-2 ELISA kit (indicated in red). Activity of IL-2 in the supernatants was measured using the CTLL-2 activity assay. RFUs normalized to non-expressing bacteria are shown (indicated in blue). (**B**) The InfB solubility tag was cloned to the N or C-terminus of the cytokine. Supernatants were collected and IL-2 signal was measured by ELISA (indicated in red). Activity of IL-2 in the supernatants was measured using a CTLL-2 activity assay. RFUs normalized to non-expressing bacteria are shown (indicated in blue). Values from three biological replicates with standard deviations are shown. *The LamB strain with C-terminal InfB tag was chosen for further experiments. *RFU* relative fluorescent unit, *RBS* ribosome binding site, *Sig peptide* signal peptide, *His*_*6*_ hexahistidine tag.

Gram-negative bacteria have been explored for their ability to colonize and proliferate in the tumor microenvironment^4,5^. In this study, we chose the probiotic EcN as the platform strain to engineer the ability to deliver IL-2 directly in the tumor microenvironment. In EcN, SecB is the dominant secretion pathway, which performs translocation of premature unfolded proteins into the periplasm, where protein folding takes place^28^. Accordingly, we chose the SecB-dependent signal peptide of OmpA as our reference strain, as this tag has been previously used for translocation of proteins into the extracellular environment^29^. Next, we cloned multiple SecB-signal peptides (Table 1) in the N-terminal of IL-2 (Fig. 1A). The constructs also harbored a C-terminal hexahistidine (His_6_) tag for purification and quantification. Of all the signal peptides investigated, only LamB showed an improvement in the extracellular titers of IL-2 compared to the initial construct with the OmpA signal peptide (Fig. 1A red bars).

**Table 1:** Signal peptides and solubility tags used in the optimization.

Signal peptide	Amino acid sequence	Source
OmpA	MKKTAIAIAVALAGFATVAQA	Choi and Lee^29^
G1M5	MNDLNDFLK TISLSFGFFLLL SLPTVAEA	Jonet et al. (2012)^30^
PelB	MKYLLPTAAAGLLLLAAQPAMA	Zhang et al. (2018)^31^
NSP4	MKKITAAAGLLLLAAQPAMA	Han et al. (2017)^32^
PhoA	MKQSTIALALLPLLFTPVTKA	Choi and Lee^29^
OmpF	MMKRNILAVIVPALLVAGTANA	Choi and Lee^29^
PhoE	MKKSTLALVVMGIVASASVQA	Choi and Lee^29^
OmpC	MKVKVLSLLVPALLVAGAANA	Choi and Lee^29^
Lpp	MKATKLVLGAVILGSTLLAG	Choi and Lee^29^
LamB	MMITLRKLPLAVAVAAGVMSAQAMA	Choi and Lee^29^
OmpT	MRAKLLGIVLTTPIAISSFA	Choi and Lee^29^
InfB solubility tag	TDVTIKT	Hansted et al.^34^
His_6_ tag	SGSGHHHHHH	This study

While protein titers are a good measure to drive strain optimization, they do not necessarily correlate with the activity of the protein expressed. Therefore, measuring protein activity is necessary to determine the amount of functional protein that is being produced. IL-2 activity was measured with an activity assay using CTLL-2 cells, which determines active IL-2 levels in a dose dependent manner. Interestingly, we observed that the protein levels of IL-2 did not correlate with the activity levels determined with CTLL-2 cells (Fig. 1A blue bars). While OmpC demonstrated low titers of IL-2, it retained the same biological activity as the reference OmpA strain. Conversely, the use of LamB signal peptide led to a ~ 50% increase in IL-2 titers; however, the activity of the protein did not increase in the same manner. These findings suggest high variability in the amount of biologically active IL-2 produced in the supernatant from the different expression constructs. Furthermore, the strains exhibited different metabolic burden, which did not correlate with the amount of produced IL-2 in the media (Supplementary Fig. S1A).

We speculated that the low titers of IL-2 may have been a consequence of the aggregation of misfolded IL-2 into inclusion bodies—a common problem when expressing heterologous proteins in *E. coli*^33^. Therefore, six strains were selected to improve protein folding and activity of IL-2 produced by different signal peptides (OmpA, LamB, OmpF, OmpC, PhoA and OmpT). We cloned the InfB solubility tag^34^ at the N- or C-terminus of IL-2 (Fig. 1B). The addition of the InfB solubility tag generally led to higher titers of IL-2 production in the supernatant. The strain OmpA with C-terminal InfB showed the highest improvement, with an eightfold increase in total IL-2 protein levels in the supernatant (Fig. 1B red bars). OmpC and LamB strains with N-terminal InfB also exhibited higher levels of IL-2, with a 3.5- and fivefold increase respectively. While OmpA with C-terminal InfB solubility tag had the highest amount of total IL-2, it showed low levels of IL-2 activity, indicating that most of the produced cytokine is inactive (Fig. 1B blue bars). Interestingly, strains that showed increased active IL-2 production were PhoA with N-terminal InfB and LamB with C-terminal InfB tag. However, the PhoA strain showed high variability in growth performance and had a higher fitness cost (Supplementary Fig. S1B). In consideration of both strain fitness and active IL-2 production, the LamB with C-terminal InfB strain was selected as the most promising candidate.

Finally, the LamB construct with C-terminal InfB solubility tag was cloned under the expression of different promoters (Table 2, Supplementary Fig. S2). We selected the MS6 and MS8 constitutive promoters as they are part of a library previously developed for consistent expression in vivo (Armetta et al. 2021^35^). Changing the promoter did not lead to any increase in IL-2 titers or activity but did impose a fitness cost on the strains (Supplementary Fig. S1C). Therefore, we continued with the strain using the Anderson promoter J23107. Overall, we chose this strain as it was able to produce high levels of functionally active IL-2 without significantly compromising the fitness of the strain. Henceforward, we refer to microbially produced IL-2 from the optimized LamB-InfB strain as mi-IL2.

**Table 2:** Promoter and RBS sequences.

Name	Sequence
J23107 Anderson promoter	TTTACGGCTAGCTCAGCCCTAGGTATTATGCTAGC
MS8 promoter	TGCTTGACTCGTCGTTATCCTACGTGTATAATTGGC
MS6 promoter	TGCTGGACTCGTCGTAATCCTGCGTGTATAATTGGC

### mi-IL2 induces strong immune cell cytotoxicity in HT29 spheroid and PBMC co-culture

**Figure 2:**
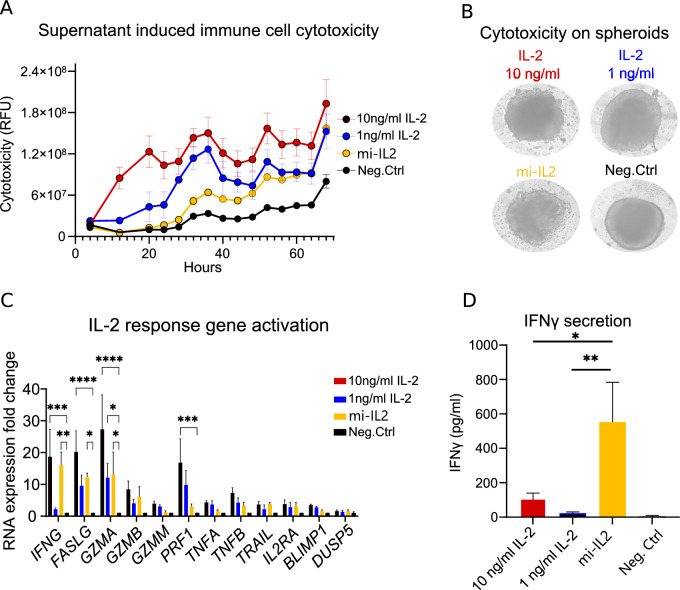
Gene expression associated with immune cell-mediated cytotoxicity is induced by the supernatant from mi-IL2. (**A**) Cytotoxicity was measured from co-cultures exposed to purified IL-2 or supernatant from mi-IL2 using Cytotox Green and the Incucyte imaging system. Three spheroids were analyzed for each condition. Supernatant from non-expressing bacteria were used as negative control (Neg.Ctrl). (**B**) Representative images of spheroid integrity after 3 days of co-culture. (**C**) RT-qPCR analysis of IL-2 response genes from tumor spheroid and PBMC co-culture. Gene expression was normalized to GAPDH expression. Mean values of relative gene expression and S.E.M. from three independent experiments are shown. qPCR primer sequences are listed in Table 3. (**D**) IFNγ levels from the tumor spheroid and PBMC co-cultures. Protein levels were measured by ELISA. Mean values of protein levels and S.E.M. from three independent experiments are shown. Significance determined with ANOVA and post-hoc analysis of comparison between groups using Tukey’s Honest Significant Difference test: (ns) p > 0.05, *p ≤ 0.05, **p ≤ 0.01, ***p ≤ 0.001.

IL-2 is a fundamental cytokine for the activation of cancer-killing immune cells, such as CD8^+^T cells and NK cells^36,37^. Thus, IL-2 promotes an immunological response by inducing the production of proteins, which participate in direct contact-mediated cytotoxicity, such as granzymes, perforin and Fas ligand (FasL)^38,39^ To determine the ability of mi-IL2 to induce an antitumor immune response, a 3D tumor spheroid assay was established. Supernatant from mi-IL2 strain was incubated with human immune cells (PBMCs) and a single HT29 tumor spheroid. mi-IL2 was added to the co-cultures at a final concentration of ~ 1 ng/ml. Purified IL-2 or supernatant from non-expressing bacteria were used as positive and negative control (Neg.Ctrl), respectively. Purified IL-2 showed a dose-dependent activation of immune cell cytotoxicity and spheroid destabilization (Fig. 2A,B). After 72 h the PBMCs exposed to mi-IL2 induced the same levels of toxicity as the purified IL-2. Furthermore, visual analysis of tumor spheroid integrity with mi-IL2 showed similar results to 10 ng/ml of IL-2 (Fig. 2B).

Next, we wanted to investigate if at a molecular level mi-IL2 would induce expression of genes associated with immune cell-mediated cytotoxicity via the same mechanism as purified IL-2. Therefore, we determined the gene expression profile in the tumor spheroid and PBMC co-cultures after 24 h exposure by RT-qPCR (primers listed in Table 3). Purified IL-2 showed a dose-dependent upregulation of genes-associated with immune cell killing (Fig. 2C). 10 ng/ml of IL-2 significantly increased expression of *IFNG*, *FASLG*, *GZMA* and *PRF1*, whereas exposure to 1 ng/ml of purified IL-2 led to lower expression levels of these genes. Interestingly, co-cultures exposed to mi-IL2 led to a strong gene activation signature similar to the 10 ng/ml of purified IL-2. This was unexpected, as the total concentration of mi-IL-2 was approximately 10× lower, but cells displayed a higher upregulation of target genes. Overall, mi-IL2 seems to promote stronger target gene activation than would be expected based on its concentration.

**Table 3:** qPCR primers.

Forward primer name	Sequence	Reverse primer name	Sequence
Human_GAPDH_fw	CGCTCTCTGCTCCTCCTGTT	Human_GAPDH_rw	CCATGGTGTCTGAGCGATGT
Human_IFNγ_fw	GAGTGTGGAGACCATCAAGGAAG	Human_IFNγ_rw	TGCTTTGCGTTGGACATTCAAGTC
Human_BLIMP1_fw	CAGTTCCTAAGAACGCCAACAGG	Human_BLIMP1_rw	GTGCTGGATTCACATAGCGCATC
Human_GrA_fw	TCTCTCTCAGTTGTCGTTTCTCT	Human_GrA_rw	GCAGTCAACACCCAGTCTTTTG
Human_GrB_fw	TACCATTGAGTTGTGCGTGGG	Human_GrB_rw	GCCATTGTTTCGTCCATAGGAGA
Human_GrM_fw	ACACCCGCATGTGTAACAACA	Human_GrM_rw	GGAGGCTTGAAGATGTCAGTG
Human_Perforin_fw	GGCTGGACGTGACTCCTAAG	Human_Perforin_rw	CTGGGTGGAGGCGTTGAAG
Human_FasL_fw	GGTTCTGGTTGCCTTGGTAGGA	Human_FasL_rw	CTGTGTGCATCTGGCTGGTAGA
Human_TnfB_fw	ACACCTTCAGCTGCCCAGACTG	Human_TnfB_rw	TCCGTGTTTGCTCTCCAGAGCA
Human_TNFa_fw	CCTCTCTCTAATCAGCCCTCTG	Human_TNFa_rw	GAGGACCTGGGAGTAGATGAG
Human_TRAIL_fw	CAGAGGAAGAAGCAACACATT	Human_TRAIL_rw	TGATGATTCCCAGGAGTTTATTTTG
Human_CD-25_fw	ACGGGAAGACAAGGTGGAC	Human_CD-25_rw	TGCCTGAGGCTTCTCTTCAC

Having seen increased *IFNG* gene expression upon stimulation with mi-IL2, we wanted to determine the levels of the gene product, interferon-γ (IFNγ), in the supernatant of the co-cultures. IFNγ is an important cytokine for immunologically mediated antitumor responses. IFNγ can directly affect tumor cells by exerting antiproliferative and anti-angiogenic effects, as well as indirectly by increasing macrophage tumoricidal activity and attracting other immune cells^40^. We quantified IFNγ levels in the conditioned media from the co-culture of PBMCs and tumor spheroids. In accordance with the gene-expression results, IFNγ levels increased with purified IL-2 stimulation in a dose-dependent manner (Fig. 2D). However, cells stimulated with mi-IL2 produced three times more INFγ, compared to 10 ng/ml of purified IL-2. These results indicate that mi-IL2 has a stronger immune-cell activating effect.

### Treatment with EcN-mi-IL2 reduces the growth of tumors in vivo

**Figure 3:**
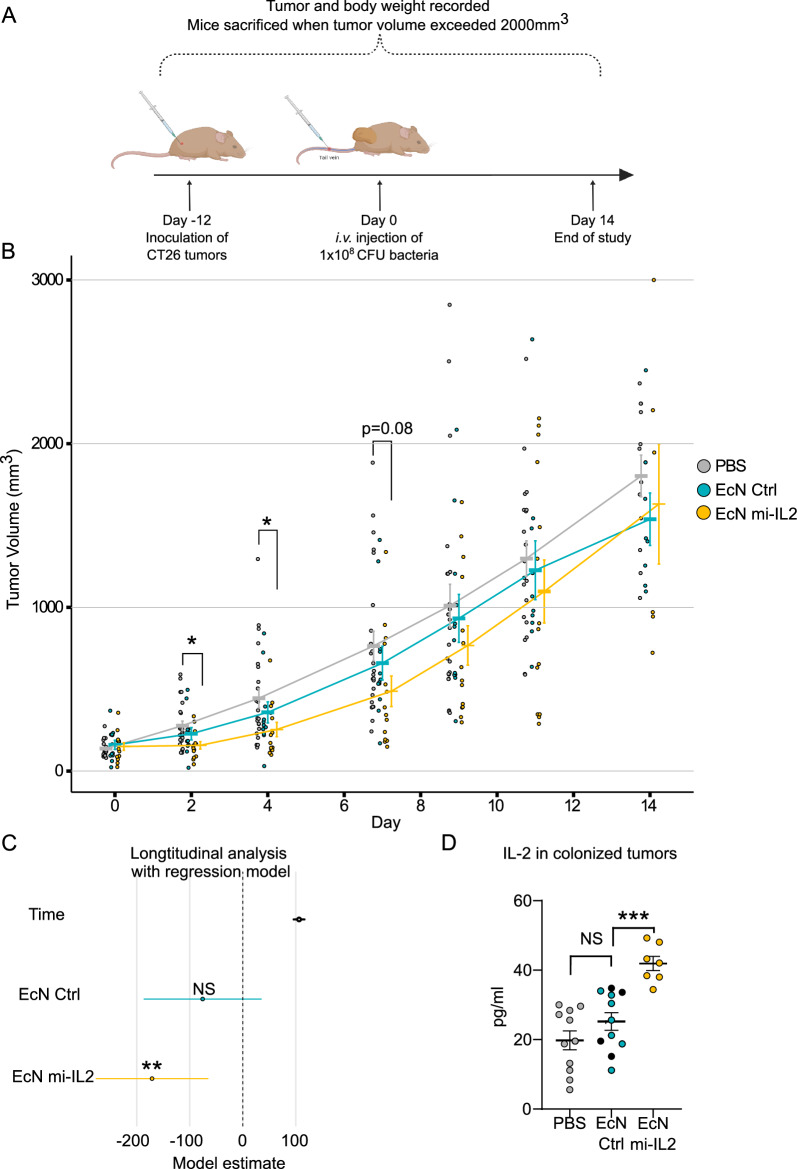
EcN mi-IL2 slows down the growth of CT26 tumors. CB6F1 mice harboring CT26 tumors on both flanks were treated with PBS or 1 × 10^8^ CFU of bacteria intravenously. Mice were sacrificed when tumors reached 2000 mm^3^. (**A**) Study design. Figure made using biorender under the agreement Number YB25KBCY7U. (**B**) CT26 tumor volumes of all mice treated with PBS (n = 12), EcN Ctrl (n = 6) and EcN mi-IL-2 (n = 8). Mean tumor volume with SEM is shown. Significance determined with ANOVA and post-hoc analysis of comparison between groups using Tukey’s Honest Significant Difference test: (ns) p > 0.05, *p ≤ 0.05. (**C**) Longitudinal analysis of growth rate of tumors using a linear regression model. The 95% confidence interval of the coefficient estimates are shown. The interpretation of the model output is the change in rate of growth of tumors when compared to PBS group. (**D**) IL-2 levels in colonized tumors with mean and SEM shown.

The antitumor effect of EcN strain expressing mi-IL2 (EcN mi-IL2) was investigated in tumor-bearing CB6F1 mice. Mice engrafted with CT26 tumors were treated with a single intravenous injection of PBS, non-expressing EcN bacteria (EcN Ctrl) or EcN mi-IL2 (Fig. 3A). Although no strong antitumor effect was observed, cross sectional analysis indicated that mice treated with EcN mi-IL2 had smaller tumors at day 2 and day 4 (Fig. 3B). Longitudinal analysis with a linear regression model indicated slower tumor growth of animals treated with EcN mi-IL2, but not the EcN Ctrl group (Fig. 3C).

Previous experiments showed that the EcN mi-IL2 strain had minimal metabolic burden, compared to WT EcN; therefore, we expected that the tumor colonization efficiency would be similar to the EcN Ctrl strain. However, we observed lower colonization of the EcN mi-IL2 compared to the EcN Ctrl. All tumors were colonized in the EcN Ctrl group, while only half of the tumors had detectable bacteria in the EcN mi-IL2 group (Supplementary Fig. S3).

Production of mi-IL2 in the tumor microenvironment is important to observe a therapeutic effect. Tumors that had bacterial colonization were homogenized and the levels of IL-2 were measured. IL-2 levels were measured from all PBS tumors and colonized tumors from EcN Ctrl and mi-IL2 groups. As expected, IL-2 levels between PBS and EcN Ctrl groups were not different (Fig. 3D), while IL-2 levels were significantly higher in tumors from the ECN mi-IL2 group, with a ~ twofold increase in concentration of intra-tumoral IL-2.

To assess whether EcN mi-IL2 had an impact on the immune cell composition of the CT26 mouse model, we performed a phenotyping analysis on immune cells isolated from spleens and tumors (Antibodies listed in Table 4). There were no differences in B or NK cell population between groups in spleen neither in tumors (Supplementary Fig. S4A,B). However, CD4^+^ T cell numbers in tumors were higher in both EcN Ctrl and EcN mi-IL2 groups, compared to PBS (Supplementary Fig. S4B). Furthermore, there was a decrease in CD8^+^ T cells in the EcN Ctrl group. Increased levels of CD4^+^ central-memory T cells and effector-memory T cells were seen in spleens from EcN mi-IL-2 group (Supplementary Fig. S4C). In the tumors, CD4^+^ T cell levels were similar between PBS and EcN mi-IL-2 groups, but were decreased in EcN Ctrl group (Supplementary Fig. S4D). CD8^+^ T cell differentiation was not different between groups in spleen and tumors (Supplementary Fig. S4E,F).

**Table 4:** Antibody mixes for immuno-phenotyping.

Antibody	Fluorochrome	BD catalog number	Dilution
CD11b	BV786	740,861	100
CD45.2	A700	560,693	50
CD3	BUV-395	563,565	20
CD4	BV-605	563,151	200
CD8	BUV-737	564,297	100
CD19	PerCp/Cy5.5	551,001	50
NKp46	BV-711	740,822	25
CD44	FITC	553,133	200
CD62L	BV-510	563,117	200

## Discussion

Bacteria engineered for local production of bioactive compounds is a promising approach for cancer treatment^41–43^, in particular for cytokines that are too toxic for systemic administration. However, in certain cases it can be challenging to express therapeutic levels of biologically active cytokines in bacteria^44,45^. Correct protein folding and post-translational modifications are crucial to retain their biological activity. Therefore, we optimized a therapeutic strain of EcN for in situ production and delivery of biologically active IL-2. Modifications of the signal peptide and solubility tag were necessary to obtain higher levels of active IL-2 (Fig. 1). We used a cell-based assay with CTLL-2 cells to determine IL-2 activity in the supernatants. In each step of the optimization, total IL-2 production was compared to the activity levels using the in vitro assay. Our results show that higher IL-2 production does not necessarily increase the amount of active IL-2 in the supernatant. This could be explained by a bottleneck in correct folding or disulphide bond formation, which results in more protein that is inactive. These findings highlight the importance of fine-tuning the expression levels in therapeutic microbes to deliver biologically active proteins.

In our study, we employed SecB-dependent signal peptides to facilitate the translocation of proteins into the periplasm. Although this approach is commonly used for extracellular protein production, the precise mechanism underlying the movement of mi-IL2 into the extracellular environment remains elusive. Previous studies have reported that proteins carrying these signal peptides are able to localize outside of the cell in a SecB-dependent manner^29,46^. While bacterial lysis emerges as a plausible pathway for mi-IL2 to access the extracellular environment, we did not observe notable adverse effects on the growth or fitness of the mi-IL2 strain when compared to the EcN control (Supplementary Fig. S1). Future studies to optimize the microbial delivery of therapeutic peptides and proteins will require a better understanding of the precise mechanism by which proteins are translocated into the extracellular environment.

An additional consideration when engineering a therapeutic microbe is the fitness and viability of the strain. In a therapeutic microbe, the resources that could be used for growth are redirected for production of heterologous proteins, which increases the metabolic burden. In turn, this can result in slower growth and lower fitness^47^. The decreased fitness can impede the ability of bacteria to survive and colonize inside the host and in the tumor microenvironment. Therefore, in this work we took into consideration the fitness cost of the strain during the optimization process (Supplementary Fig. S1), with the aim to balance the levels of heterologous bioactive protein and the fitness cost on the strain.

Using our 3D cell culture assay we were able to show that microbial IL-2 is more potent than the purified protein. The same target genes were upregulated in mi-IL2 and purified IL-2 (Fig. 2C), indicating, that the mechanism of action is IL-2-dependent. However, mi-IL2 showed a stronger effect in activation of immune cells than purified IL-2, even though the protein levels were tenfold lower. Conditioned media from bacteria not only contains IL-2, but also other immunogenic molecules derived from bacteria, like lipopolysacharide (LPS) that can act as an adjuvant. LPS has been reported to work synergistically with IL-2 in enhancing IFNγ production and cytotoxicity^48^. Therefore, the additive effect of bacterially produced IL-2 together with LPS and other immunogenic molecules could improve the therapeutic response.

Treatment with EcN mi-IL2 did not have a strong therapeutic effect in mice bearing CT26 tumors. Some tumors treated with EcN mi-IL2 did not respond to the treatment and this resulted in higher variability. Nevertheless, slower tumor growth in early time points and higher levels of IL-2 in the tumors were observed (Fig. 3). It has been previously reported that *E. coli* TOP10 and EcN infection of BALB/c mice bearing CT26 tumors leads to a CD8^+^ T cell-mediated tumor clearance^24,49^. In our CB6F1 mouse model, we saw a decrease of intratumoral CD8^+^ T cells in mice treated with non-expressing EcN (EcN Ctrl), whereas the mice from EcN mi-IL2 group had the same levels of total CD8^+^ T cells as the PBS group (Supplementary Fig. S4B). It is possible that in our mouse model CD8^+^ T cells are not activated upon infection with bacteria as observed by the modest therapeutic effect of the strain. Finally, cytokine delivery into the tumor microenvironment will be impacted by low tumor colonization, thus affecting the therapeutic efficacy of the treatment. We hypothesized, that a strain with a better growth profile would result in stable colonization of the tumor microenvironment. For instance, EcN Ctrl strain exhibited very stable colonization of tumors throughout the whole experiment. In contrast, EcN mi-IL2 strain exhibited decreased tumor colonization (Supplementary Fig. S3), even though the growth profile was similar to the EcN Ctrl strain (Supplementary Fig. S2). Unfortunately, growth profiling in the laboratory cannot fully recreate the conditions, which influence bacterial survival in the tumor microenvironment. Furthermore, expression of immune cell-activating cytokines might also have an impact on bacterial colonization. For example, it has been reported that macrophage bactericidal activity against *S. typhimurium* was increased upon IL-2 stimulation^50^. Perhaps the cytokine-expressing bacteria promote their own elimination by activating macrophages or other bacteria-killing cells. It is not clear whether the poor tumor colonization is due to the metabolic burden caused by cytokine expression, or the immunological impact of the cytokine. Further research is needed to identify the specific effects causing this phenomenon, but repeated bacterial administration could be a potential solution.

Intratumoral IL-2 delivery has been studied previously with *S. typhimurium* with promising preclinical results^21^. However, *S. typhimurium* producing IL-2 did not have a significant effect in clinical trials^51^. Another caveat is that *S. typhimurium* is a pathogenic bacterium, which requires attenuation^52^. Other bacterial chassis have also been explored for IL-2 delivery, including *Clostridium* species, however in those studies efficacy for treating cancer in vivo was not demonstrated^22,23^. Furthermore, these studies did not optimize for highest IL-2 activity in bacterial supernatants, nor for the optimal fitness cost of the strain. As we have shown in our work, higher total IL-2 production outside of the cell does not necessarily result in more active IL-2. Furthermore, different signal peptides or addition of solubility tags not only change active IL-2 production in the supernatant, but also the fitness cost of the strain. Therefore, we argue that strains should be optimized for highest IL-2 activity and not for total IL-2 levels found in the supernatants.

In this work we demonstrate the feasibility of EcN for delivery of IL-2 in situ as a potential immunotherapy treatment. Although IL-2 has been expressed in *E. coli* before, to our knowledge this is the first work, which demonstrates intratumoral delivery of IL-2 for cancer therapy. However, further optimization is needed in order to achieve a stronger therapeutic effect. Future applications may include further optimization of the strains, more complex genetic circuits using biosensors^53^ or co-expression of other therapeutic peptides that could exert a synergetic anticancer effect. Although the road to clinical applications still poses several challenges, this work will benefit the society by bringing us closer to safer and more targeted delivery of therapeutics to treat diseases in a transformative and target-oriented manner.

## Materials and methods

### Cloning of cytokine-expressing strains

The native *E. coli* Nissle 1917 plasmid pMut1 was used for expression of the cytokines. The plasmid has been previously modified to insert a Kanamycin resistance gene for antibiotic selection. Plasmid backbone was PCR amplified with Phusion high fidelity PCR master mix with primers, which encoded the relevant, promoter, signal peptide or solubility tag (Tables 1, 2). The PCR fragments would produce 20–25nt overhangs with the DNA sequences coding the cytokines. DNA sequences coding the cytokines were taken from UniProt. IL-2 gene was codon optimized for *E. coli* and synthesized by IDT. The constructs were cloned into the pMut1 plasmid using Gibson assembly according to manufacturer’s recommendations. After Gibson assembly, the plasmids were transformed into *E. coli* TOP10 strain either using electro competent or chemically competent cells. Correct strains were selected by colony PCR and Sanger sequencing. Plasmids were purified from TOP10 bacteria and were transformed into electro competent *E. coli* Nissle 1917 bacteria to produce the final cytokine-expressing strain.

### Preparation of bacterial supernatants

The media was supplemented with 10% FBS and 50 μg/ml of Kanamycin. RPMi media was used for making supernatants for IL-2 activity assay. McCoy’s 5A media was used for making supernatants for 3D tumor spheroid cytotoxicity assay. A single bacterial colony was inoculated into RPMi or McCoy’s 5A media and grown overnight at 37 °C with shaking 250 RPM. Overnight cultures were diluted 100-times into fresh media and grown until late exponential phase. Cultures were centrifuged at 4000×*g* for 10 min and supernatants were filter-sterilized using a 0.22 μm syringe filter. Supernatants were stored at − 20 °C for further use.

### Quantification of cytokines in supernatants

Bacterial or cell culture supernatants were diluted in ELISA dilution buffer and cytokine levels were measured with a commercial ELISA kit (Abcam ab46033) according to the manufacturer’s recommendations.

### Cell culture

Mammalian cells were maintained in T75 flasks and subcultured every 3–4 days. Complete growth media contained 10% FBS and 1% antibiotic cocktail. CTLL-2 cells (RRID:CVCL_0227) were cultured in complete RPMi media supplemented with 10% T-cell culture supplement with ConA (Thermofisher Scientific 11513540). CT-26 cells (RRID:CVCL_7254) were cultured in complete RPMi media. HT29 cells (RRID:CVCL_A8EZ) were cultured in complete McCoy’s5A media. The cells were grown in a humidified incubator at 37 °C with 5% CO2 atmosphere.

### CTLL-2 activity assay

CTLL-2 cells were centrifuged at 200G for 5 min and washed twice with PBS to remove the IL-2 culture supplement from the media. The cells were starved for IL-2 by incubating them in complete RPMi media without any IL-2 for 5–6 h. 2 × 10^4^ cells in a 45 μl of volume were placed into each well of a 96-well black, clear-bottom plate. 45 μl of diluted supernatants were added to the cells at a final concentration 10% (V/V). Media with 5% of T-cell culture supplement with ConA was used as a positive control, because it contains IL-2. After 16 h 10 μl of Alamar blue (Thermofisher Scientific, DAL1025) was added and incubated for 30 min. Fluorescence intensity was measured at 530 nM excitation and 560 nM of emission wavelengths.

### PBMC isolation

Fresh human buffy coats were obtained from healthy anonymized volunteers at the blood bank of the Department of Clinical immunology, Rigshospitalet, Copenhagen, Denmark. PBMCs were isolated by density gradient centrifugation using Lymphoprep and SepMate-50 tubes. Isolation was performed according to manufacturer’s recommendations. Frozen PBMCs were stored for later use. PBMCs were used immediately after thawing.

### 3D tumor spheroid cytotoxicity assay

5000 HT-29 cells were seeded in a 96-Well Clear Ultra Low Attachment Microplate (Thermofisher Scientific, 10023683) and centrifuged at 1000×*g* for 10 min and were placed into the incubator to form spheroids. 4-day old tumor spheroids were co-cultured with 1 × 10^5^ human PBMCs and supernatants from bacteria (30% V/V) in a volume of 100 μl. Human recombinant IL-2 was used for positive control (Thermofisher Scientific, PHC0026). 1 μl of 30× diluted Cytotox green dye (Essen Biosciences, ESS4633) was added per well and cytotoxicity was measured up to 72 h using the Incucyte S3 live cell imaging system. Spheroid integrity images were taken after 72 h of incubation. Co-cultures were gently pipetted up/down 5 times to disturb the pelleted cells and pictures were acquired immediately with the Incucyte system.

### Analysis of target gene expression in PBMC monoculture and tumor spheroid and PBMC co-culture

Human PBMCs or tumor spheroid + PBMCs were incubated with bacterial supernatants or recombinant IL-2 (Thermofisher Scientific, PHC0026) for 24 h. The assay was performed using 12 wells of a 96-Well Clear Ultra Low Attachment Microplate for each condition. Cells were pooled into a 1.5 ml Eppendorf tube and the cells were pelleted by centrifugation at 200×*g* for 10 min. The supernatant was frozen and used to assay INFγ secretion with a commercial ELISA kit (Thermofisher Scientific, 02002) and the pelleted cells were resuspended in TRIzol. RNA was extracted (Zymo research, R2072) and reverse-transcription reaction (Thermofisher Scientific 11756050) was performed according to manufacturers’ recommendations. cDNA was diluted 6-times with MiliQ water. 3-step qPCR reactions were performed by mixing 6 μl of the 2× qPCR master mix (Merck, KK4602), 2ul of cDNA and 2 μl of qPCR primers (3 μM) (Table 3).

### Animal experiments

All animal experiment protocols were conducted according to the Danish guidelines for animal welfare. The animal experiment council at the Danish animal experiments inspectorate has approved the study protocols with the experimental license nr. 2017-15-0201-01209. The study was carried out in accordance to the ARRIVE guidelines. CB6F1 mice were bought from Envigo. CT26 cells were washed 3 times in PBS and 5 × 10^5^ cells were inoculated into each flank of the mice. Sizes of the tumors were measured and the volume was calculated according to the formula $\frac{3.14}{6}\times {D1\times D2}^\frac{3}{4}$, where D1 and D2 are dimensions in millimeters. After 10–12 days when tumors have formed and reached at least 100 mm^3^ size mice were injected *i.v.* with PBS or 1 × 10^8^ CFU of bacteria in a 100 μl volume. Tumor volumes and mouse weight were measured 3 times per week. Mice were sacrificed when tumors reached 2000 mm^3^ volume. Due to rapid growth of some tumors, some mice exceeded the 2000 mm^3^ limit upon inspection and were sacrificed immediately. Mice were killed by cervical dislocation and tumors and organs were dissected. Dissected tumors were cut into small pieces and divided into two tubes to analyze tumor colonization-cytokine levels and immuno-phenotyping.

### Assessing colonization and cytokine levels in tumors and organs

After dissection even-sized parts of the tumors, half of the spleen or whole liver were placed into GentleMACS C tubes and homogenized using the m_Imptumor_01_01 program. Cell clumps were removed by forcing the homogenized samples through a 70 μm cell strainer. Serial dilutions of the organ homogenates were plated on LB plates containing Kanamycin and bacterial CFUs were counted the next day. To quantify expressed cytokine levels in the tumors—the same organ homogenates were centrifuged at 5000×*g* for 10 min at 4 °C. The supernatant was transferred to a new tube and centrifuged at 17,000×*g* for 15 min at 4 °C. The supernatant was stored at − 80 °C. ELISA was performed with twofold diluted supernatants to measure IL-2 in the tumors. ELISA (Abcam ab46033) was performed according to manufacturer’s recommendations.

### Preparation of tumor and spleen cells for immuno-phenotyping

For tumor digestion 50 μl of DNAase I (Sigma-Aldrich, 11284932001) (stock 600 U/ml) and 0.5 ml of collagenase IV (Thermofisher Scientific, 2525) (stock 10 mg/ml) were incubated with small pieces of tumor in complete DMEM in a total 5 ml volume. The digestion was carried out at 37 °C for 45 min with thorough shaking. Spleens were homogenized with GentleMACS C tubes as discussed above. Cell clumps were removed by forcing the homogenized samples through a 70 μm cell strainer. The cells were washed with PBS 3 times and cells were frozen at 1 × 10^7^ viable cells per cryotube. The media was supplemented with 10% DMSO and RPMi media was used for spleens and DMEM media for tumor cells.

### Immuno-phenotyping of tumors and spleens

Frozen cells from tumors or spleens were quickly thawed with warm complete RPMi media and immediately transferred into a 15 ml falcon tube with 9 ml of warm RPMi media. The cells were centrifuged at 1500RPM for 5 min at 4 °C and washed with selection buffer (1× PBS, 2% FBS, 1 mM EDTA). Cells were filtered through a filter FACS tube and CD45 positive selection (StemCell Technologies, 18945) was performed only on the tumor cells according to manufacturer’s recommendations. After selection, cells were washed with FACS buffer (1× PBS, 2% FBS) and transferred to a 96-well plate. Cells were incubated with 10 μl Fc block on ice for 10 min. After the incubation the cells were washed with FACS buffer and incubated with the antibody panel (Table 4) for 30 min on ice. Cells were washed twice with FACS buffer and fixed with 50 μl PFA overnight at 4 °C. The next day cells were washed with FACS buffer twice and filtered through a filtering plate before running on the flow cytometer. Gating strategy is indicated in Supplementary Fig. S5.

## Supplementary Information

Supplementary Figures.

## Data Availability

Flow cytometry data is available at FlowDepository database. Flow cytometry files for spleen immunophenotyping can be accessed following the link http://flowrepository.org/id/FR-FCM-Z6KG. Flow cytometry files for immunophenotyping in the tumor can be accessed following the link http://flowrepository.org/id/FR-FCM-Z6KH. Other data is available upon request to the corresponding authors.
