# Supplementary material for: Engineered E. coli Nissle 1917 for delivery of bioactive IL-2 for cancer immunotherapy
Source: Sci Rep. 2023 Aug 2;13:12506. doi: 10.1038/s41598-023-39365-2 (PMC10397246; doi:10.1038/s41598-023-39365-2)
Supplement: Supplementary file 1 — Supplementary Figures. [file 41598_2023_39365_MOESM1_ESM.pdf]

Engineered *E.coli* Nissle 1917 for delivery of bioactive IL-2 for cancer immunotherapy

Sarunas Tumas[1], Trine Sundebo Meldgaard[2], Troels Holger Vaaben[1], Sara Suarez Hernandez[2], Annemette Tengstedt Rasmussen[1], Ruben Vazquez-Uribe[1], Sine Reker Hadrup[2], Morten O.A. Sommer[1,*]

Supplementary information

Affiliations:

[1] Novo Nordisk Foundation Center for Biosustainability, Technical University of Denmark, Lyngby, Denmark

[2] Department of Health Technology, Technical University of Denmark, Lyngby, Denmark

* Corresponding author: msom@bio.dtu.dk

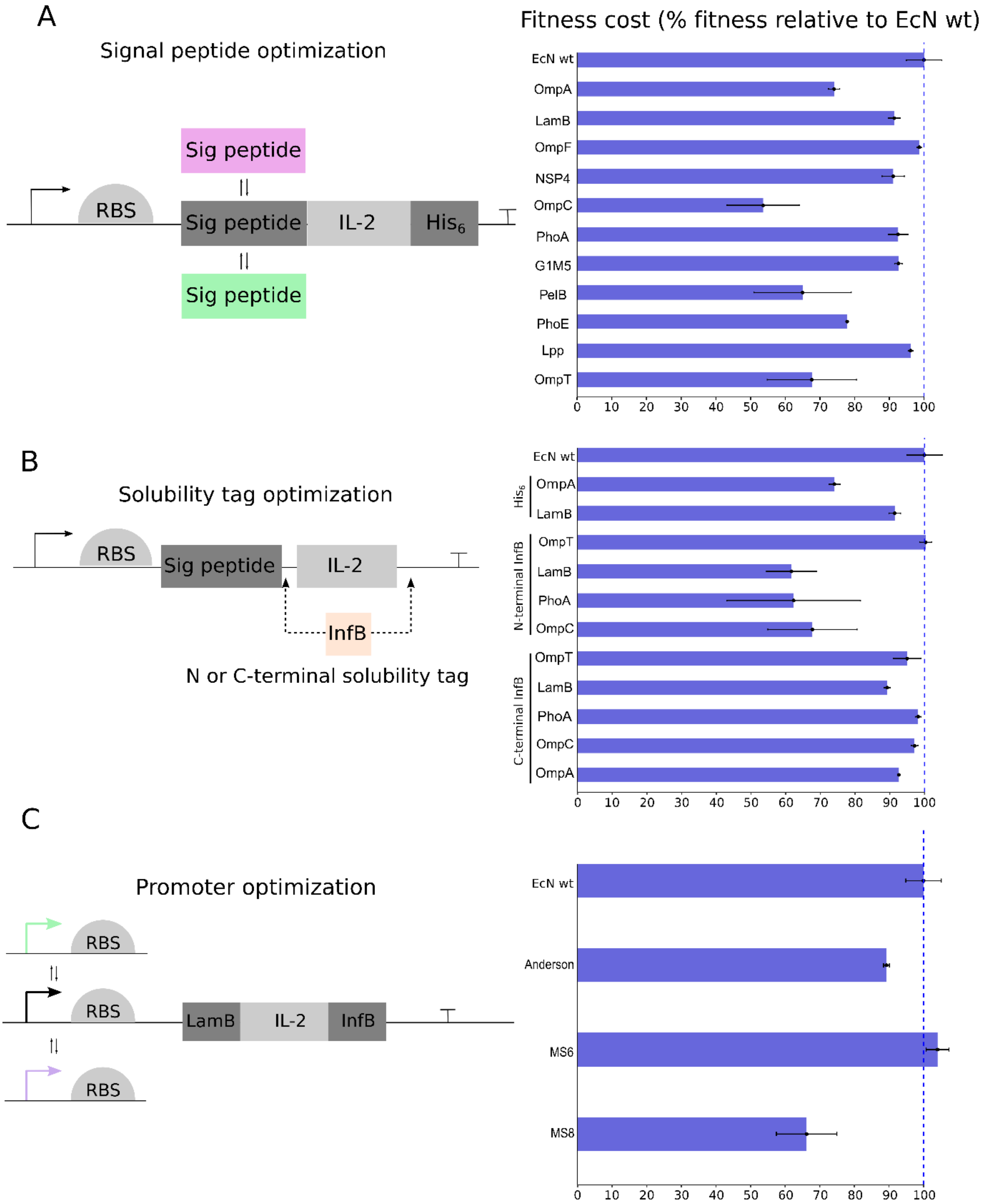

Figure S1. Fitness cost of developed strains. S1. Mean relative fitness cost (in %) compared to non-expressing EcN calculated from biological triplicates. **A)** strains used in figure 1A. **B)** strains used in figure 2A. **C)** Optimization of the promoter for the LamB-InfB IL-2 strain. RBS – ribosome binding site; Sig peptide – signal peptide; His_6 – Hexahistidine tag, EcN wt – Wild type *E.coli* Nissle 1917.

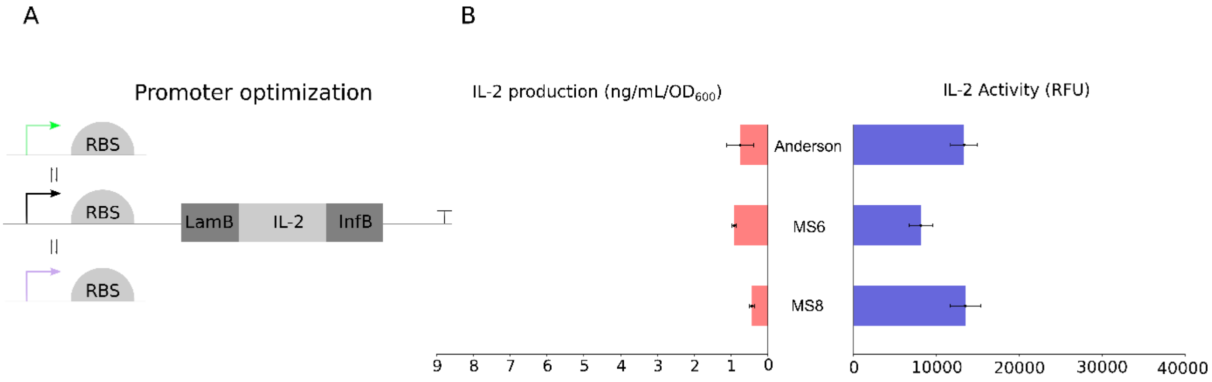

Figure S2. Promoter optimization of mi-IL2. A) MS6 and MS8 promoters were clones upstream of the mi-IL2 construct. The MS8 promoter is stronger than the previously used Anderson promoter and MS6 is of similar strength to the previously used Anderson promoter. **B)** Supernatants were collected and IL-2 was measured by ELISA (indicated in red). Activity of IL-2 in the supernatants was measured using a CTLL-2 activity assay (indicated in blue). RFUs normalized to non-expressing bacteria are shown. Values from three biological replicates with standard deviations are shown. RFU – relative fluorescent unit; RBS – ribosome binding site.

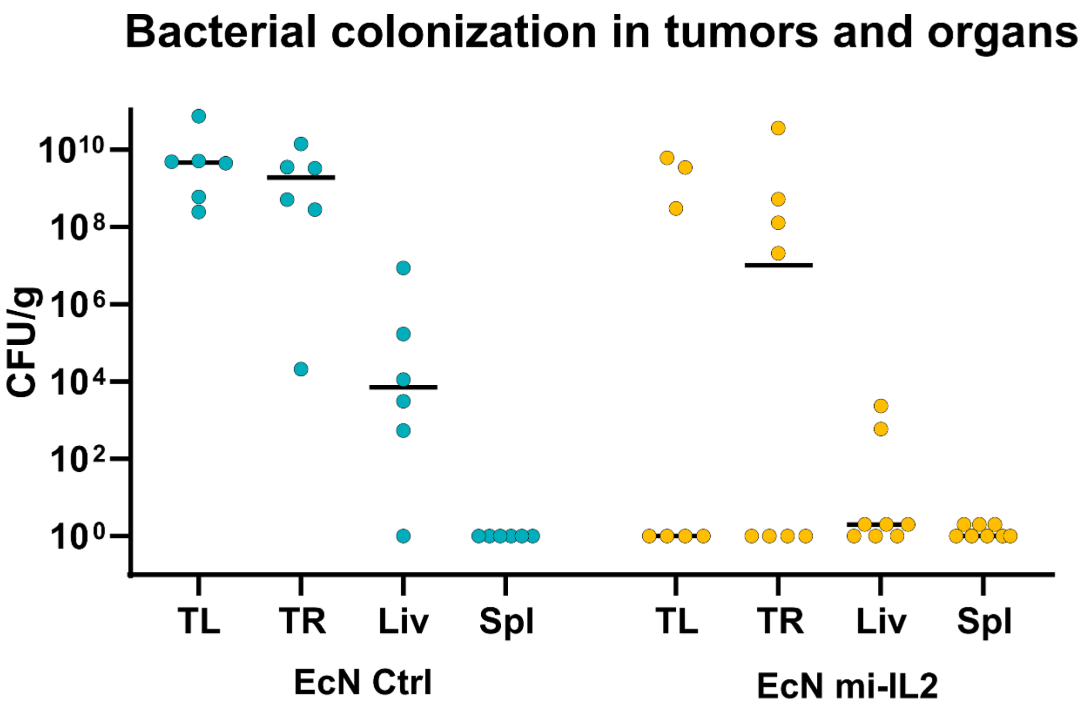

Figure S3. Tumor and organ colonization in mice. Tumors and organs were homogenized and serial dilutions were plated on LB plates. Individual values of each tumor and the median is shown.

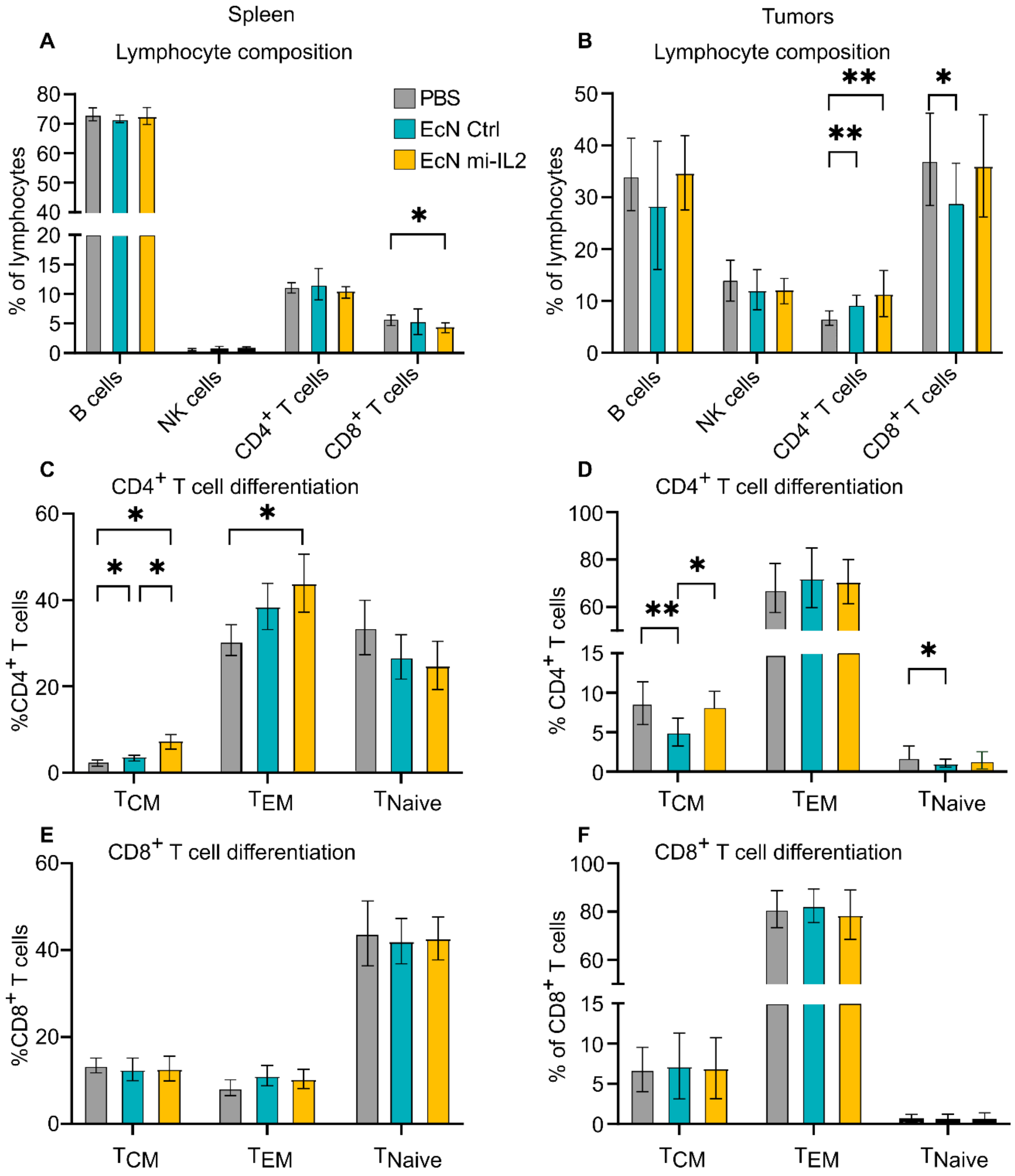

Figure S4. Bacteria producing IL-2 shift CD4+ T cell differentiation to central memory type in spleens and tumors. Analysis of immune cell composition was performed from tumors and spleens from the mice treated with PBS (n=12), non-expressing EcN Ctrl (n=6) and EcN mi-IL-2 (n=8). The organs were homogenized, stained with cell-specific antibodies and analysed by flow cytometry. A - B) Immune cell composition of lymphocytes (CD45+ CD11bcells) in spleens and tumors. CD19 and CD335 were used to gate B and NK cells, respectively. CD3+CD4+ cells were labelled as CD4+ and CD3+CD8+ cells were labelled as CD8+ . C-D) Differentiation of CD4+ T cells in spleens and tumors into centralmemory T cells (TCM; CD44highCD62high), effector-memory T cells (TEM ;CD44highCD62L-) and naïve T cells (TNaive;CD44-CD62Lhigh). E-F) Differentiation of CD8+ T cells in spleens and tumors with the same parameters as for CD4+ T cells. Means and S.E.M are plotted. Oneway ANOVA was performed to assess statistical significance. Only significant differences are shown. TCM – T central-memmory; TEM – T effector-memmory.

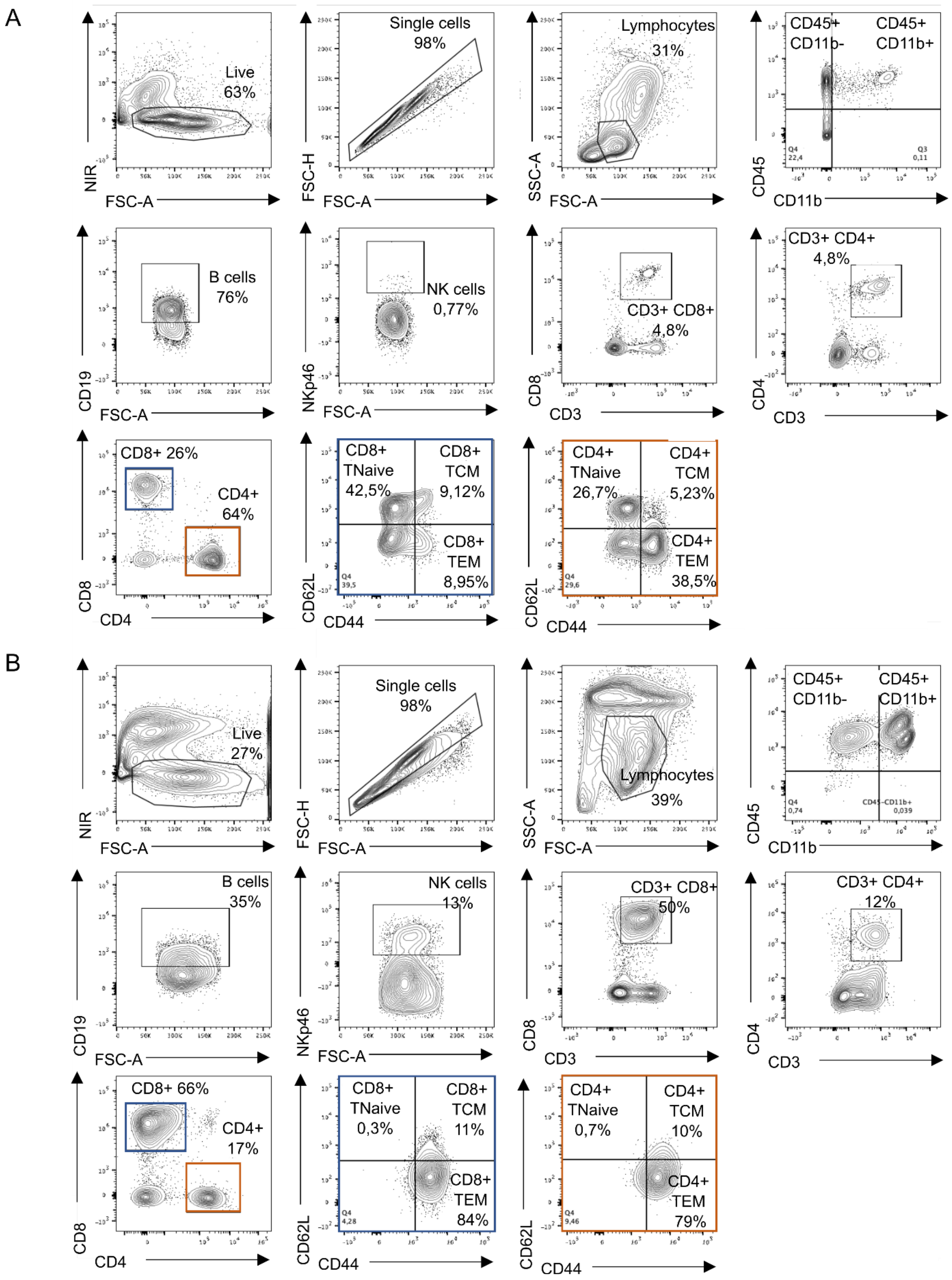

Figure S5. Gating strategy. **A)** Gating strategy of spleens. **B)** Gating strategy of tumors. Representative plots from an EcN mi-IL2 treated mouse. B, NK, CD8^{+} and CD4^{+} were gated out of the CD45+CD11b-. Blue (CD8^{+}) and orange (CD4^{+}) squares represent the parent population of the T cell differentiation plots.
